# Kv channel-interacting proteins as neuronal and non-neuronal calcium sensors

**DOI:** 10.1080/19336950.2018.1491243

**Published:** 2018-08-02

**Authors:** Robert Bähring

**Affiliations:** Institut für Zelluläre und Integrative Physiologie, Zentrum für Experimentelle Medizin, Universitätsklinikum Hamburg-Eppendorf, Hamburg, Germany

**Keywords:** Ca^2+^, calsenilin, DREAM, KChIP, Kv4 channel, NCS protein, recoverin

## Abstract

Kv channel-interacting proteins (KChIPs) belong to the neuronal calcium sensor (NCS) family of Ca^2+^-binding EF-hand proteins. KChIPs constitute a group of specific auxiliary β-subunits for Kv4 channels, the molecular substrate of transient potassium currents in both neuronal and non-neuronal tissues. Moreover, KChIPs can interact with presenilins to control ER calcium signaling and apoptosis, and with DNA to control gene transcription. Ca^2+^ binding via their EF-hands, with the consequence of conformational changes, is well documented for KChIPs. Moreover, the Ca^2+^ dependence of the presenilin/KChIP complex may be related to Alzheimer’s disease and the Ca^2+^ dependence of the DNA/KChIP complex to pain sensing. However, only in few cases could the Ca^2+^ binding to KChIPs be directly linked to the control of excitability in nerve and muscle cells known to express Kv4/KChIP channel complexes. This review summarizes current knowledge about the Ca^2+^ binding properties of KChIPs and the Ca^2+^ dependencies of macromolecular complexes containing KChIPs, including those with presenilins, DNA and especially Kv4 channels. The respective physiological or pathophysiolgical roles of Ca^2+^ binding to KChIPs are discussed.

## Introduction

“KChIP” stands for “Kv channel-interacting protein”, a name that was chosen by An and coworkers [1] at the turn of the century, certainly not to proclaim a novel discovery of proteins that interacted with voltage-gated potassium channels, given that a number of such auxiliary proteins had been known previously [2]. Rather, these authors most specifically searched for interaction partners with a yeast two hybrid bait corresponding to the cytoplasmic N-terminus (180 amino acids) of the Kv4.3 subtype of Kv4 channels. These channels are related to the *Shal* gene of *Drosophila melanogaster* [3,4], and mediate both the subthreshold-activating somatodendritic A-type current (I_SA_) in neurons [5,6] and the transient outward current (I_to_) in cardiomyocytes [7–9]. The search for Kv4 channel auxiliary β-subunits had been driven by the finding that coexpression of a low molecular weight brain mRNA fraction (2 – 4 kb) caused an increase in surface expression and a modification of the biophysical properties of *Shal*-related A-type (i.e., rapidly inactivating) channels [10,11], and, in fact, heterologous coexpression of the newly identified KChIPs had very similar effects on Kv4 channels [1]. It is now widely accepted that KChIPs, together with dipeptidyl aminopeptidase-like proteins (DPPs), are integral components of native A-type potassium channels in neurons and cardiomyocytes [5,8,12,13].

KChIPs belong to the neuronal calcium sensor (NCS) family, sometimes also referred to as the recoverin superfamily, of Ca^2+^-binding proteins [14]. The KChIP subfamily itself has four members: KChIP1, KChIP2, KChIP3 and KChIP4, with multiple splice variants known for each subtype [1,15–17]. When the KChIPs were initially described [1], it became immediately clear that one of them, KChIP3, is virtually identical to the previously and independently discovered proteins calsenilin [18] and DREAM (for “downstream regulatory element antagonist modulator”) [19]. The fact that calsenilin or DREAM also interacted with Kv4 channels defined a third function for the very same protein, indeed justifying the name “KChIP”, at least within the NCS protein family. The finding that the newly identified members of the NCS family modulated the surface expression and biophysical properties of Kv4 channels, gave rise to the intriguing idea that “KChIPs may regulate Kv4 channel-mediated currents, and hence neuronal excitability, in response to changes in intracellular Ca^2+^” [1]. However, the role of KChIPs as specific and tightly associated Ca^2+^ sensors for Kv4 channels, although highly attractive and intriguing, has been rarely approached experimentally [20–22]. This review article will first give a brief overview of the NCS protein family. Then current knowledge on the Ca^2+^ binding properties of KChIPs will be summarized and the physiological or pathophysiological implications of Ca^2+^ binding for KChIP complexes with presenilins, DNA and Kv4 channels will be discussed.

## The NCS protein family

**Figure 1.**
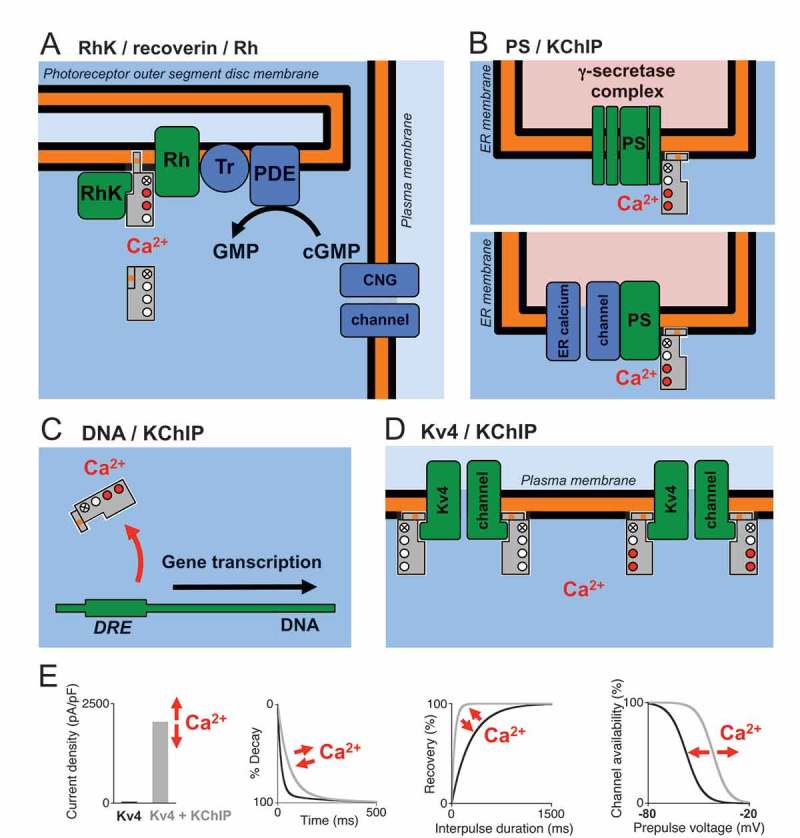
**Ca^2+^ sensor function of recoverin and KChIPs**. The NCS proteins (grey rectangles with EF-hands depicted as circles) and their target molecules (green) are shown (indirect targets in blue). Putative or mechansitically proven Ca^2+^ dependences (red filled circles: EF-hands occupied by Ca^2+^) are indicated, but no reference is made to the oligomerization state of KChIPs. (A) Recoverin exhibits a Ca^2+^/myristoyl switch, allowing complex formation with both rhodopsin kinase (RhK) and rhodopsin (Rh) and anchoring of the complex in the disc membrane. Recoverin prevents phosphorylation (i.e., inhibtion) of Rh by RhK, which augments the cascade involving the G-protein transducin (Tr) and a phosphodiesterase (PDE) to efficiently convert cyclic guanosine monophosphate (cGMP) into GMP and eventually close cyclic nucleotide-gated (CNG) channels. (B) KChIP3 (calsenilin) interacts with presenilins (PS) to control the γ-secretase complex (γ-cleavage of APP protein, upper panel), and/or to control ER Ca^2+^ channels (lower panel). (C) KChIP3 (DREAM) binds to the downstream regulatory element (DRE) sites of genes when Ca^2+^ is low, to suppress their transcrption. Ca^2+^-dependent dissociation of KChIP3 (DREAM) from the DNA allows the transcription of those genes. (D) KChIPs associate with Kv4 channels to augment their surface expression and to modulate their inactivation gating. The Ca^2+^ dependence of Kv4/KChIP complex formation seems to depend on Kv4 and/or KChIP subtype, trafficking seems to be Ca^2+^-dependent, and acute modulation of membrane-bound Kv4/KChIP complexes by changes in cytoplasmic Ca^2+^ concentrations has not been studied in structural detail. (E) Graphs show the effects of Kv4/KChIP coexpression (black: Kv4 alone, grey: Kv4 + KChIP). Shown data (increase in current density, acceleration of initial curent decay kinetics, acceleration of recovery from inactivation and positive shift of the voltage dependence of steady-state inactivation) refer to Kv4.2 + KChIP2c coexpression [70]. Red arrows indicate that Ca^2+^ binding to KChIPs may have an influence on the observed effects.

**Figure 2.**
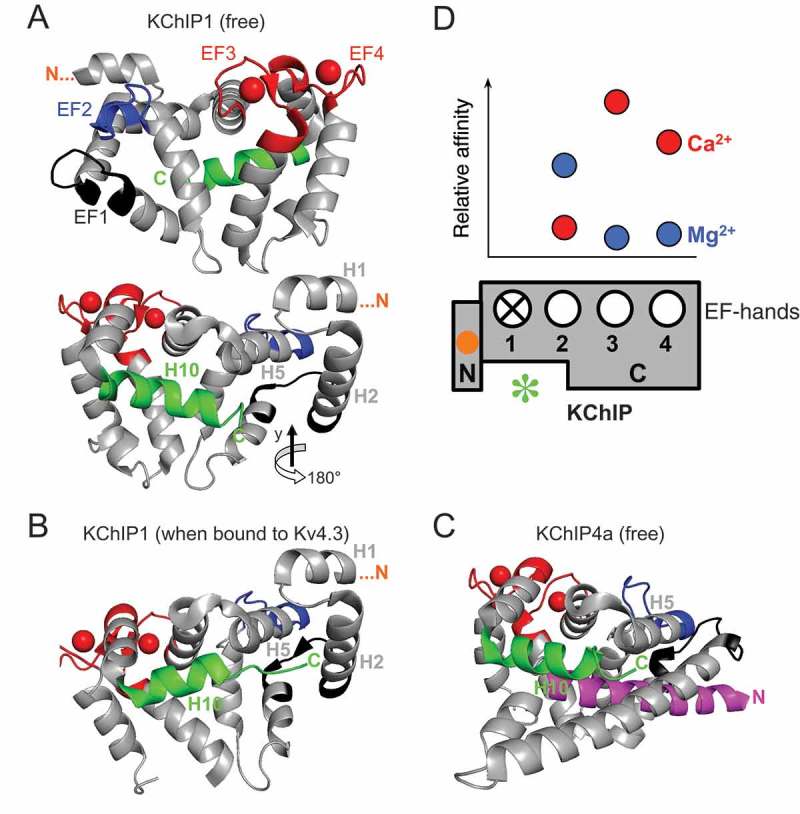
**KChIP structure and calcium sensor function**. Important structural determinants of KChIP function and EF-hand-mediated divalent cation binding affinities. (A) Crystal structure of the free KChIP1 monomer (PDB 1S1E) [31] viewed from two different sides. Upper picture: N-terminus on the left (structure starts at position 38) and EF-hands visible; EF1 (black) is non-functional, and EF2 (blue) is occuopied by Mg^2+^ under physiological conditions. Two Ca^2+^ ions (red spheres) reside in EF3 and EF4, respectively (red). Lower picture: Rotating the KChIP1 structure by about 180° around the vertical axis (N-terminus on the right) offers a better view of the H10 α-helix (green), which lines the hydrophobic groove. (B) Crystal structure of a KChIP1 molecule when bound to Kv4.3 (PDB 2I2R) [30]. Positioning according to the N-terminal H1 and H2 α-helices of the lower picture in A suggests conformational changes, especially of the C-terminal H10 α-helix which is moved aside for target molecule binding. (C) Crystal structure of the free KChIP4a molecule (PDB 3DD4) [83]. Positioning according to the H5 α-helix in A (lower picture) and B reveals that the KChIP4a structure substantially differs from KChIP1. Notably, the hydrophobic groove is lined by the KChIP4a N-terminus (magenta), known to contain the KIS domain and an ER retention signal. (D) Relative binding affinities for Ca^2+^ (red) and Mg^2+^ (blue) were estimated from the K_d_ values for divalent cation binding of KChIP2c single EF-hand mutants [37] and plotted in relation to the respective EF-hands in a schematic representation of the KChIP molecule. The KChIP N-terminus is a functionally relevant structural entity (orange dot: myristoylation or palmitoylation site; KIS domain not indicated); green asterisc: hydrophobic groove.

The NCS proteins are functionally diverse, being involved in the control of retinal photoreceptor sensitivity, neurotransmitter release, ion channel trafficking and function, gene transcription, as well as neuronal growth and survival [14]. Ion channels, known to be either directly or indirectly regulated by NCS proteins, include Kv4 and Kv1 channels, voltage-dependent Ca^2+^ (Cav) channels, cyclic nucleotide-gated (CNG) channels, as well as α-amino-3-hydroxy-5-methyl-4-isoxazolepropionic acid (AMPA) type glutamate, nicotinic acetylcholine (nACh) and purinergic (P2X) receptors [14,23], but also endoplasmic reticulum (ER) Ca^2+^ release channels like the ryanodine and the inositol trisphosphate (IP3) receptor (see section “KChIPs interact with presenilins”). The following proteins, assigned to different classes (A – E), according to their first appearance in evolution and/or to meet functional differences [14], belong to the NCS family (see also Table 1): Frequenin (also called NCS-1) represents class A; hippocalcin, neurocalcin-δ and the visinin-like proteins (VILIPs) represent class B; recoverin represents class C; guanylyl cyclase-activating proteins (GCAPs) represent class D; and the KChIPs represent class E. There are four different KChIP subtypes: KChIP1, KChIP2, KChIP3 (also known as calsenilin or DREAM) and KChIP4 (also known as CALP for “calsenilin-like protein”). The NCS proteins are about 200 – 250 amino acids long, they have a conserved core domain and variable N- and C-termini. Notably, the N-terminus of NCS proteins may play a critical role in post-translational modification and protein function: Proteins in classes A – D and the class E protein KChIP1 possess an N-terminal myristoylation sequence, and myristoylation of these proteins allows their membrane association [24,25]. Moreover, recoverin (class C) and all class B members of the NCS family exhibit a so-called Ca^2+^/myristoyl switch, meaning that the N-terminal myristoyl moiety, which is sequestered in a hydrophobic groove in the Ca^2+^-free state, swings out when the protein binds Ca^2+^ [26,27]. In recoverin, which is a calcium sensor *par excellence*, this Ca^2+^-dependent conformational change allows the interaction with its target proteins (rhodopsin kinase and rhodopsin), and, at the same time, the association of the protein complex with the photoreceptor outer segment disc membrane (Figure 1A) to control light sensitivity in a Ca^2+^-dependent manner [28]. Certain KChIP2, KChIP3 and KChIP4 splice variants may be N-terminally palmitoylated to favor membrane association [14,29], and in the KChIP4a splice variant the N-terminus harbors a K channel inactivation suppressor (KIS) domain (see section “Ca^2+^ dependence of Kv4/KChIP complex formation and membrane trafficking”) [14,15]. The conserved NCS core domain contains four EF-hand motifs (EF1, EF2, EF3 and EF4), a feature known from calmodulin, but in all NCS proteins the most N-terminal EF-hand motif (EF1) is degenerated and cannot bind Ca^2+^[14]. Recoverin [26], KChIP1 [30–32] and KChIP3 [33] were even isolated with only two Ca^2+^ ions bound (see Figure 2 and next section). The Ca^2+^ sensitivity of the NCS proteins is very high. Ca^2+^ binding occurs slightly above 100 nM in a cooperative manner, and the binding affinity lies well above the one of calmodulin [14,24].

**Table 1.** The NCS protein family.

				Ion channel targets	
Class	Protein(s)	Subtypes	Other name(s)	direct interaction	indirect regulation
A	Frequenin		NCS-1	Cav, Kv4	
B	Hippocalcin				AMPAR
	Neurocalcin-δ				CNG
	VILIPs	VILIP1		nAChR, P2XR	CNG
		VILIP2		Cav	
		VILIP3			
C	Recoverin				CNG
D	GCAPs	GCAP1			CNG
		GCAP2			CNG
		GCAP3			CNG
E	KChIPs	KChIP1		Kv4, Kv1	
		KChIP2		Kv4, Kv1, Cav	Kv4, Nav, Cav, RyR
		KChIP3	Calsenilin, DREAM	Kv4	Cav, IP3R
		KChIP4	CALP	Kv4	

The NCS proteins are assigned to classes A – E. Ion channel targets were largely adapted from Burgoyne [14] and from Burgoyne and Haynes [23], but some are based on newer findings for KChIP2 and KChIP3 [54,57,58,69]. AMPAR, α-amino-3-hydroxy-5-methyl-4-isoxazolepropionic acid receptor; CALP, calsenilin-like protein; Cav, voltage-dependent calcium (channel); CNG, cyclic nucleotide-gated (channel); DREAM, downstream regulatory element antagonist modulator; GCAP, guanylyl cyclase-activating protein; IP3R, inositoltrisphosphate receptor; KChIP, Kv channel interacting protein; Kv, voltage-dependent potassium (channel); nAChR, nicotinic acetylcholine receptor; NCS, neuronal calcium sensor; P2XR, purinergic receptor; RyR, ryanodine receptor; VILIP, visinin-like protein.

## Ca^2+^ binding properties and associated conformational changes of the KChIPs

The binding of divalent cations to KChIPs has been studied using wild-type and EF-hand-mutated (ΔEF) proteins. The experimental results showed that KChIPs are able to bind both Ca^2+^ and Mg^2+^ via their EF-hands, and that they exhibit high and low affinity Ca^2+^ binding (Figure 2) [1,34–39]. K_d_ values for high affinity Ca^2+^ binding range between 10 and 50 μM, and for low affinity Ca^2+^ binding between 2 and 3 mM [35,37,38]. High affinity Ca^2+^ binding is distorted with ΔEF3 or ΔEF4, but not with ΔEF2, which leads to a drop in Mg^2+^ affinity [34,35,37]. Three Ca^2+^ ions may bind, with descending affinity, to EF3, EF4 and EF2, however, in the presence of Mg^2+^ both wild-type and ΔEF2 KChIPs have been shown to bind only two Ca^2+^ ions [39]. Taken together, these findings suggest that in KChIPs EF2 mediates low affinity Ca^2+^ binding and is occupied by Mg^2+^ under physiological conditions, whereas EF3 and EF4 mediate high affinity Ca^2+^ binding [34–37,39]. The data are in accordance with crystal structure and NMR analysis, which showed only two bound Ca^2+^ ions per KChIP subunit residing in EF3 and EF4 (Figure 2) [30–33]. Obviously, EF3 and EF4 are critical sites for a putative calcium sensor function of the KChIPs.

KChIPs undergo conformational changes in a divalent cation-dependent manner. Ca^2+^ withdrawal from KChIP1 [30], KChIP2 [38] and KChIP3 [39] has been shown to cause a reduced helical content and increased flexibility of these proteins. Even an EF3,EF4 fragment (amino acid 161 – 256) of KChIP3 showed a reduced spectral resolution in the absence of Ca^2+^, corroborating the central role of EF3 and EF4 in Ca^2+^ binding and associated conformational changes [40]. KChIPs may also form oligomers in a divalent cation-dependent manner, with Ca^2+^ binding to EF3 and EF4 favoring dimer formation [33,39,41]. KChIP3 has even been reported to oligomerize with calcineurin and calmodulin [42]. Thus, the diversity of complex formation between KChIPs and their target molecules (see below) may be further increased by the oligomerization of KChIPs with a number of closely and more distantly related proteins [42]. KChIPs have three main types of specific target molecules, and three corresponding functions are attributed to the KChIPs: Some of them have been shown to interact with presenilins to control Ca^2+^ signaling and apoptosis (Figure 1B), and some have been shown to interact with DNA to control gene expression (Figure 1C), but all KChIPs interact with Kv4 channels (Figure 1D) to form I_SA_ and control neuronal excitation [5,12]. Moreover, due to the expression of KChIP2 as a critical component of the Kv4 channel-mediated I_to_ in cardiomyocytes [8,43,44], it is obvious that KChIPs also play an important role in non-neuronal excitation. To which extent EF-hand-mediated Ca^2+^ binding plays a role in any of the functions attributed to KChIPs shall be discussed in the following.

## KChIPs interact with presenilins

One type of specific target molecule for KChIPs is represented by the presenilins (PS1 and PS2), intracellular membrane-associated proteins (Figure 1B), mutations in which are related to early-onset familial Alzheimer’s disease [45]. The first KChIP to be identified as an interaction partner for both PS1 and PS2 was KChIP3, then dubbed “calsenilin”, because it was able to bind both calcium and presenilin [18]. KChIP3 (calsenilin) was identified in a yeast two-hybrid screen using a 103 amino acid C-terminal fragment (CTF) of PS2 as a bait. With the same method, but using a 43 amino acid CTF of PS2, KChIP4 was later identified as another presenilin interaction partner, and dubbed CALP for “calsenilin-like protein” [16]. KChIP3 (calsenilin) and KChIP4 (CALP), both diffusely distributed in the cytoplasm when expressed alone, were shown to be targeted to intracellular membranes when co-expressed with PS2 [16,18]. Presenilins are part of a multi-protein γ-secretase enzyme complex, which mediates the γ-cleavage of amyloid precursor protein (APP) to produce Aβ peptides, a process which is dysregulated in Alzheimer’s disease [45]. Since KChIP3 (calsenilin) overexpression enhances this γ-secretase activity, it has been suspected that KChIP3 (calsenilin) represents a calcium sensor of the γ-secretase enzyme complex (Figure 1B) [46]. Binding assays have shown that the interaction between KChIP3 (calsenilin) and a 23 amino acid CTF of PS1 is highly Ca^2+^-dependent, with a more than 200-fold decrease in protein binding affinity (K_d_ of 183 μM instead of 0.6 μM) in the absence of divalent cations [47]. Moreover, the data suggested that, in the presence of Ca^2+^, dimeric KChIP3 (calsenilin) may bind two presenilin molecules [47].

Aside from its Ca^2+^-dependent γ-cleavage-associated function, the presenilin/KChIP complex plays a role in controlling intracellular calcium signaling itself, since presenilins may directly interact with and facilitate the function of both IP3 and ryanodine receptors in the ER (Figure 1B) [48–51]. Leissring and coworkers found that the abnormally increased amplitude and accelerated decay of IP3-mediated Ca^2+^ transients, observed in the presence of mutant PS1, returned to normal when KChIP3 (calsenilin) was co-expressed. This effect was specifically based on the presenilin/KChIP3 (calsenilin) interaction, and not due to KChIP3 (calsenilin)-mediated Ca^2+^ buffering, because KChIP3 (calsenilin) expression alone did not influence Ca^2+^ transients [52]. Taken together, KChIP3 (calsenilin) with its target proteins PS1 and PS2 plays a critical role in both APP γ-cleavage and ER Ca^2+^ release, functions which are dysregulated in Alzheimer’s disease [53]. Physiological aspects and pathophysiological maladaptations of the presenilin/KChIP3 (calsenilin) interaction will have to be mechanistically dissociated more clearly in the future.

More recent studies in cardiomyocytes suggest that, similar to KChIP3 (calsenilin), KChIP2 may also influence ryanodine receptor-mediated Ca^2+^-induced Ca^2+^ release, by interacting with presenilins [54]. It was observed that KChIP2 antisense knock-down led to a decreased amplitude and a broadening of Ca^2+^ transients, associated with a reduced sarcomeric shortening. These effects were not due to compromized sarcoplasmic reticulum (SR) Ca^2+^ loading, and correlated with a dispersion of PS1 from SR membranes to the cytoplasm [54]. Surprisingly, the effects on Ca^2+^ transients caused by KChIP2 knock-down [54] resembled the ones previously observed with KChIP3 (calsenilin) overexpression [52]. Structural differences between KChIP2 and KChIP3 and corresponding differences in their Ca^2+^ binding properties, but also the different cell systems used in these studies may be responsible for the seemingly contradictory findings. Certainly, more research is needed to elucidate the KChIP/presenilin-mediated control of Ca^2+^-induced Ca^2+^ release.

## KChIPs control gene transcription

There are numerous examples showing that KChIPs are involved in the control of gene expression in both excitable and non-excitable cells. Especially KChIP3, but also KChIP1, KChIP2 and KChIP4, may fulfill the function of a transcriptional repressor, either directly, by binding to downstream regulatory element (DRE) sites of genes [19,55–61], or indirectly, by binding to other transcription factors [62–64]. Here the focus is put on direct KChIP-mediated control of gene transcription (Figure 1C), which appears to be commonly linked to excitability. KChIP3 is identical to a previously isolated Ca^2+^-dependent transcriptional repressor, then dubbed “downstream regulatory element antagonist modulator” (DREAM) [19]. When Ca^2+^ is low KChIP3 (DREAM) was shown to specifically bind to the DRE site of the human prodynorphin gene, thereby preventing its transcription. An increase in Ca^2+^ leads to conformational changes and a dissociation of KChIP3 (DREAM) from the DRE site in an EF-hand-dependent manner, which allows transcription of the prodynorphin gene [19]. Thus, in nociceptive spinal cord neurons the prodynorphin gene is switched on during massive action potential firing with elevated cytoplasmic Ca^2+^ levels, to modulate pain perception via κ-opioid receptors [19,41,65]. In an analogous manner the Na^+^/Ca^2+^ exchanger (NCX) 3 expression in cerebellar neurons is under the control of the transcriptional repressor KChIP3 (DREAM), which may occupy a doublet of DRE sites in the NCX3 gene when Ca^2+^ is low [55]. Dissociation of KChIP3 (DREAM) from these DRE sites in an EF-hand-dependent manner and activation of the NCX3 gene occurs if cytoplasmic Ca^2+^ is elevated, which has been interpreted as a negative feed-back control loop of Ca^2+^ homeostasis [55]. KChIP3 seems to also exist as a cytosolic protein which is translocated to the nucleus if Ca^2+^ signaling is out of control [66]. Intriguingly, cytosolic KChIP3 (DREAM) has been detected in cardiomyocytes and shown to be translocated to the nucleus in a Ca^2+^- and Ca^2+^/calmodulin-dependent protein kinase (CaMK) II-dependent manner. In the nucleus KChIP3 (DREAM) represses the transcription of the CACNA1c gene, which codes for the L-type Ca^2+^ channel α-subunit Cav1.2 [58]. Apparently, in cardiomyocytes CaMKII activity inverts the otherwise releasing effect of elevated Ca^2+^ on the transcription repressor function of KChIP3 (DREAM), but in the end also serves Ca^2+^ homeostasis. For KChIP2, despite its prominent expression in cardiomyocytes, a gene transcription control mechanism similar to the ones involving KChIP3 (DREAM) could not be shown [67]. Rather, KChIP2 has been reported to up-regulate I_Ca_ via a direct interaction with the Cav1.2 protein independently of protein expression or trafficking (see section “Acute modulation of the Kv4/KChIP channel complex by cytoplasmic Ca^2+^”) [68,69]. Notably, however, it was recently found that in cardiomyocytes KChIP2 acts as a transcritional repressor for certain micro RNAs (miR-34b and miR-34c). If those are expressed during cardiac stress, which is often associated with a loss of KChIP2, these micro RNAs may target ion channel genes, including SCN5A, SCN1B and KCND3, to suppress the synthesis of the sodium channel α-subunit Nav1.5, the Navβ1-subunit and the potassium channel Kv4.3, respectively [57]. These results reveal a novel role for KChIP2 in cardiac disease states including arrhythmias and cardiac remodeling. The Ca^2+^ and EF-hand dependence of the KChIP2-mediated control of miR-34b/c expression remains to be studied mechanistically in more detail.

## Ca^2+^ dependence of Kv4/KChIP complex formation and membrane trafficking

KChIPs are specific β-subunits of Kv4 potassium channels (Figure 1D). the molecular substrate of transient potassium currents in heart and brain [5,8,12]. Heterologous coexpression of KChIPs (except for the KChIP4a splice variant, see below) leads to an increase in surface expression of Kv4 channels and to a modulation of their inactivation gating, namely, a slowing of the initial phase of macroscopic current decay, an acceleration of the recovery from inactivation and a positive shift of the voltage dependence of steady-state inactivation (Figure 1(e)). [1,70,71] As for any other type of NCS protein and its target molecule(s), a putative calcium sensor function of KChIPs related to Kv4 channels [1] may be mediated in various, not mutually exclusive, ways: One possibility is that Kv4/KChIP complex formation itself depends on the Ca^2+^ binding state of the KChIP β-subunit. This aspect also includes the intriguing possibility of a Ca^2+^-dependent dynamic Kv4:KChIP stoichiometry. Also, trafficking of Kv4/KChIP complexes to the plasma membrane or its internalization may depend on Ca^2+^ binding to the KChIP β-subunit. All of these putative mechanisms are expected to modify the molecular composition and/or the number of Kv4 channels in the plasma membrane. Alternatively, or in addition to that, membrane-bound Kv4/KChIP complexes may be preserved when Ca^2+^ binds to the KChIP β-subunit, however, conformational changes of the KChIPs may be directly transmitted to the channel in order to modulate its gating (Figure 1D,E). As described above, Ca^2+^-dependent complex formation undoubtedly underlies the function of recoverin in retinal photoreceptors and, in a reverse manner, the DNA/KChIP-mediated control of gene expression, but probably also the presenilin/KChIP-mediated control of APP γ-cleavage and ER Ca^2+^ release. The reports on the role of EF-hand-mediated Ca^2+^ binding in Kv4/KChIP complex formation are conflicting: Pioletti and coworkers, who studied the interaction between Kv4.3 and KChIP1 in great detail, found that neither a KChIP1ΔEF2,3,4 nor a KChIP1ΔEF3,4 mutant was able to modulate Kv4.3 channel inactivation gating when coexpressed [30]. These results correlated well with their pull-down experiments showing that for both KChIP1ΔEF2,3,4 and KChIP1ΔEF3,4 binding to a 143 amino acid N-terminal fragment (NTF) of Kv4.3 was impaired, and the authors concluded that Ca^2+^ binding to KChIP1 EF-hands is required for Kv4.3/KChIP1 complex formation [30]. Likewise, the triple EF-hand mutant ΔEF2,3,4 of the KChIP4b1 splice variant (the initially identified CALP) was not able to modulate gating and did not coimmunoprecipitate with Kv4.2 [16], and the binding of KChIP3 to a Kv4.3 152 amino acid NTF has been shown to be Ca^2+^-dependent [72]. An and coworkers [1] showed that the KChIP1ΔEF2,3,4 mutant was unable to modulate the inactivation gating of Kv4.2, however, these authors reported (without showing the data) that KChIP1ΔEF2,3,4 coimmunoprecipitated with the Kv4.2 α-subunit, supporting the notion that Kv4.2/KChIP1 complex formation is not Ca^2+^-dependent. Apparently, this applies also to Kv4.2/KChIP2 complex formation, because Bähring and coworkers [70] found that binding of KChIP2c to a 180 amino acid NTF of Kv4.2 occurs both in the absence and presence of Ca^2+^. Structural differences between Kv4.3 and Kv4.2, differences in KChIP binding efficiency between NTFs and Kv4 full-length protein, and, not least, structural differences between different KChIP subtypes, may be responsible for the seemingly contradictory results. Also, depending on the amount of KChIP available, the number of KChIPs bound to a Kv4 channel may vary [73]. However, nothing is known about the Ca^2+^ dependence of the variable Kv4:KChIP stoichiometry, nor is it known whether this applies to membrane bound Kv4/KChIP complexes. The latter would be an intriguing way to regulate Kv4 channel function. Definitely, more research is needed to elucidate the role of Ca^2+^ in Kv4/KChIP complex formation. In this regard it should also be noted that both the Kv4 N-terminus and the Kv4 tetramerization (T1) domain contribute to KChIP binding [31,74], and each KChIP subunit interacts with the N-terminus of one and the T1-domain of a neighboring Kv4 α-subunit [30,75]. It is possible that the Ca^2+^ dependencies of KChIP binding to these two sites differ. In support of this notion, it was recently found that, with strong cytoplasmic Ca^2+^ buffering by BAPTA-AM starting immediately after cell transfection, KChIP2b coexpression caused an acceleration of Kv4.3 recovery kinetics, but not the otherwise typical slowing of the initial phase of macroscopic current decay [22]. Accordingly, coexpression of KChIP2bΔEF2 or KChIP2bΔEF4 in the presence of Ca^2+^ markedly accelerated Kv4.3 recovery kinetics but did not considerably slow macroscopic current decay [22]. These data comply well with the results obtained by Patel and coworkers, who studied a minimal KChIP2d splice variant cloned from ferret heart, which consists only of the final 70 amino acids of other KChIP2 splice variants [76]. Notably, coexpression of KChIP2d, which possesses only one EF-hand (EF4), caused the typical effects on Kv4.3 macroscopic current decay and recovery from inactivation, whereas coexpression of the KChIP2dΔEF mutant only modulated Kv4.3 recovery kinetics [76]. It is possible that Kv4.3/KChIP2 complex formation occurs in a different fashion depending on whether Ca^2+^ is bound to KChIP2 or not. Thus, in the absence of Ca^2+^ KChIP2 binding to the Kv4.3 N-terminus may not be strong enough to suppress N-type inactivation [77], while KChIP2 binding to the Kv4.3 T1-domain may still be sufficiently strong to modulate other aspects of inactivation [71,78]. Such a modulatory rather than decisive role of EF-hand-mediated Ca^2+^ binding in Kv4/KChIP complex formation was also supported by the finding that binding of wild-type KChIP2c to a 90 amino acid NTF of Kv4.2 is enhanced, whereas the binding of KChIP2cΔEF mutants to that fragment, although possible, is not enhanced by Ca^2+^ [35,37].

It has been recognized early on that, with the exception of the KChIP4a splice variant, complex formation with KChIPs promotes the trafficking of Kv4 channels to the plasma membrane and increases their stability [1,70,79]. It was suspected that some ER retention motif within the Kv4 N-terminus is masked, and thus rendered ineffective, during Kv4/KChIP complex formation [70,79]. In such a scenario Ca^2+^-sensitive KChIP binding to the Kv4 N-terminus (see above) would also confer Ca^2+^ sensitivity to channel trafficking. Hasdemir and coworkers [80] studied Kv4.2 channel trafficking, including its KChIP and Ca^2+^ dependence, in more detail: They found that Kv4.2 expressed alone is not retained in the ER but can reach the Golgi compartment, and that coexpression of KChIP1 allows further trafficking to the plasma membrane. The Ca^2+^ binding-deficient mutant KChIP1ΔEF2,3,4 was not only unable to promote membrane trafficking but even prevented Kv4.2 from reaching the Golgi compartment. Apparently, Kv4.2 interacts with KChIP1 (wild-type or mutant) somewhere along the ER-to-Golgi transport pathway in a Ca^2+^-independent manner, but Ca^2+^ binding to KChIP1 is required for further trafficking to the plasma membrane. The Ca^2+^-dependent step of Kv4.2/KChIP1 trafficking has not been identified yet, but Ca^2+^-dependent targeting of the channels to the right transport vesicles seems unlikely since KChIP1ΔEF2,3,4 and wild-type KChIP1 showed the same subcellular localization [80]. In KChIP1 and other NCS proteins the vesicular targeting information has been shown to reside in the N-terminal myristoylation motif [25], but in contrast to recoverin (Table 1A) and the class B NCS proteins (Table 1) KChIP1 does not exhibit a Ca^2+^/myristoyl switch, so the myristoyl moiety is available for membrane association already at resting Ca^2+^ concentrations [25].

The KChIP4a splice variant is special in various respects: Coexpression of KChIP4a does not lead to increased Kv4 channel surface expression [79] and leaves the voltage dependence of steady-state inactivation and the kinetics of recovery from inactivation unaffected. However, KChIP4a completely suppresses the fast macroscopic inactivation of Kv4 channels, resulting in delayed rectifier-like currents [15]. The N-terminal KIS domain of KChIP4a, which also harbors an ER retention motif, is made responsible for these trafficking and gating effects [15,81]. In the KChIP4a splice variant the hydrophobic groove, which represents a binding domain of KChIPs for the Kv4 N-terminal helix [30,75,82], can be occupied by its own N-terminal helix in vitro (Figure 2(c)), and it was suggested that it may have to be competed out by the Kv4 N-terminus to allow Kv4/KChIP4a complex formation [83,84]. With KChIP2x and KChIP3x (= KChIP3b) Jerng and Pfaffinger [85] have identified two brain KChIP isoforms, which, like KChIP4a, contain an N-terminal KIS domain. Notably, these authors found that the N-termini of the KIS domain-containing KChIPs are actually membrane-spanning in a cellular environment, which contradicts a competition with the Kv4 N-terminus [85]. Nevertheless, as we have seen, autoinhibitory binding of the N-terminus on the one hand, and membrane association of the N-terminus on the other hand, represent hallmarks of the Ca^2+^-dependent function of recoverin (Figure 1A) Like in other KChIPs, EF3 represents the high affinity Ca^2+^ binding site of KChIP4a (Figure 2), and Ca^2+^ (10 μM) increases the Kv4.3 NTF (amino acid residues 6 – 145) binding affinity of KChIP4a by an order of magnitude [86]. However, structure-function analysis on the KIS domain-containing KChIPs with respect to their interaction with Kv4 channels has not considered the role of Ca^2+^ binding.

## Acute modulation of the Kv4/KChIP channel complex by cytoplasmic Ca^2+^

Certainly, for ion channel physiologists the most intriguing scenario concerning a calcium sensor function of KChIPs consists in the notion that Kv4/KChIP complexes in the plasma membrane respond, more or less instantaneously, to fluctuations in cytoplasmic Ca^2+^ with modified gating. Chen and coworkers [87] were the first to directly examine the effects of changing the cytoplasmic Ca^2+^ concentration on voltage-dependent potassium currents. These authors managed to exchange the patch-pipette solution, in order to vary the cytoplasmic Ca^2+^ concentration, while performing whole-cell recordings from individual cultured guinea pig hippocampal neurons. They observed an increase in I_SA_ amplitude during initial cell perfusion with low Ca^2+^ and a suppression of I_SA_ during subsequent cell perfusion with high Ca^2+^. The cytoplasmic Ca^2+^ increase caused opposing and overlapping effects on I_SA_ and the delayed-recifier potassium current (I_D_), but I_SA_ suppression (> 50%) persisted if the authors applied Cs^+^ or tetraethylammonium to block I_D_ [87]. In another study, Wang and coworkers [88] used patch-pipette solutions with different Ca^2+^ concentrations in separate recordings from cultured rat cerebellar granule cells to study possible differences in I_SA_ properties. Unlike Chen et al., these authors observed almost 100% larger I_SA_ amplitudes when recording with the high Ca^2+^ patch solution, which did match their simultaneously observed slower current decay kinetics but not the more negative voltage dependence of steady-state inactivation, and no Ca^2+^ dependence at all was seen for the kinetics of recovery from inactivation [87,88]. In none of the two above-mentioned studies [87,88] a statement was made about the actual free Ca^2+^ concentration in the patch-pipette solutiuon (i.e., in the cytoplasm), which complicates proper judgement of the described effects. Nevertheless, Wang et al. reported that Co^2+^ was not capable of reproducing their Ca^2+^ effects, supporting the notion that these required a Ca^2+^-binding protein and were not caused just by increasing divalents on the internal side of the membrane [88]. Moreover, Wang et al. performed experiments in the presence of arachidonic acid [88], which is known to suppress the amplitude and accelerate the decay kinetics of Kv4 channel-mediated currents in a KChIP-dependet manner [89]. From their observation that arachidonic acid prevented the Ca^2+^ effects on I_SA_ the authors concluded that these were somehow mediated by KChIPs. Thus, the study by Wang and coworkers [88] did support the notion of an acute Ca^2+^-dependent modulation of I_SA_-related Kv4/KChIP channels. More recently, Groen and Bähring [22] examined the Ca^2+^-dependent gating modulation of Kv4.3/KChIP2 channel complexes expressed in human embryonic kidney (HEK) 293 cells. These authors accounted for the actual free Ca^2+^ concentration in the patch-pipette, and their results suggested that the kinetics of recovery from inactivation were affected by varying cytoplasmic Ca^2+^ between nominal Ca^2+^-free (BAPTA or BAPTA-AM) and 50 μM Ca^2+^. In that study Kv4.3 was not only coexpressed with wild-type but also with specifically EF-hand-mutated KChIP2, and the observed Ca^2+^ dependences were further examined in the presence of the CaMKII inhibitor KN-93. The results were explained by a mechanism in which direct Ca^2+^ binding to KChIP2 EF3 and EF4 may speed up the recovery of Kv4.3/KChIP2 channels from inactivation. However, CaMKII action may apparently override this effect, causing a net Ca^2+^-mediated slowing of Kv4.3/KChIP2 recovery kinetics with KChIP2 EF2 and EF3 being critically involved. In view of this putative mechanism the findings suggest a shift from the functional importance of both high affinity Ca^2+^ sites (EF3 and EF4) to a combined functional importance of a Ca^2+^ and a Mg^2+^ site (see also Figure 2D) of KChIP2 when CaMKII is active. Notably, in their experiments with the minimal KChIP2d splice-variant Patel et al. saw no effect on recovery kinetics when they incubated their Kv4.3/KChIP2d expressing oocytes in BAPTA-AM [76]. These results suggest that the single EF-hand (EF4) remaining in KChIP2d is not sufficient for this form of Ca^2+^-dependent gating modulation.

The above-described experimental approaches have all imposed changes in Ca^2+^ concentration to the entire cytoplasm. However, physiologically relevant sudden increases in Ca^2+^ concentration may be restricted to cytoplasmic microdomains (less than 1 μm distance) [14,90] of Ca^2+^ entry (i.e., Ca^2+^ permeable channels in the plasma membrane) or Ca^2+^ release (i.e., ER Ca^2+^ channels). In their seminal studies, Anderson and coworkers [20,21] identified Ca^2+^ entry via Cav3 channels, known to mediate a subthreshold-activating transient (T-type) Ca^2+^ current, as a possible source of Ca^2+^ for the modification of A-type channel gating. What these authors initially observed, was a negative shift of the voltage dependence of I_SA_ steady-state inactivation when they applied the T-type Ca^2+^ channel blocker mibefradil, but not when they applied the high voltage-activated (HVA) Ca^2+^ channel blocker SNX-483, to stellate cells in rat cerebellar slices [20]. Their coimmunoprecipitation results suggested the existence of a macromolecular signaling complex consisting of Kv4.2, KChIP3 and Cav3. In fact, the mibefradil results could be reproduced when these components were coexpressed, together with a DPP-subunit, in tsA-201 cells, but not if KChIP3 was replaced by KChIP1, KChIP2 or KChIP4 [20,21]. Thus, it is highly likely that Cav3 channels represent the physiological source of Ca^2+^ for an acute gating modulation of membrane-bound Kv4.2/KChIP3 channels. In fact, Heath and coworkers [91] found out that regional differences in Cav3 channel expression directly correlated with the voltage-dependent I_SA_ availability, and blocking Ca^2+^ currents abolished regional differences in I_SA_ density. The novel form of subcellular Ca^2+^ signaling occurs within a protein complex, and the radius of action actually represents a Ca^2+^ nanodomain (distance < 50 nm) [14,90]. From the results obtained with a Ca^2+^ channel blocker (negative shift of the inactivation curve) it can be inferred that Ca^2+^ entry and Ca^2+^ binding to KChIP3 causes a positive shift of the inactivation curve, which represents a strengthening of the actual KChIP effect on Kv4 channel gating (see Figure 1D,E). Thus, Ca^2+^ influx keeps the voltage dependence of steady-state inactivation in a depolarized range in order to guarantee a high I_SA_ availability at subthreshold potentials, necessary for the control of action potential firing in stellate cells [20]. When external Ca^2+^ is decreased, as during repetitive synaptic activity in the molecular layer of the cerebellum, the availability of stellate cell I_SA_ is decreased to maintain inhibitory charge transfer to Purkinje neurons [92]. It will be interesting to see whether a comparable Kv4/KChIP/Cav nanodomain signaling also exists in other neurons, as suspected recently in the case of Kv4/KChIP/DPP complexes in nociceptive DRG neurons [93], or in cardiomyocytes. Notably, in cardiomyocytes KChIP2 may form a complex with Cav1.2 [68,69], maybe in combination with Kv4 channels. Also, CaMKII, which seems to play a critical role in the Ca^2+^-dependent regulation of Kv4/KChIP channels [22], may participate in this cardiac signaling complex [94–96]. Finally, it has to be examined whether other sources of Ca^2+^, including transmitter-gated channels like N-methyl-D-aspartic acid (NMDA) receptors, or ER Ca^2+^ channels like the ryanodine and the IP3 receptor, are able to acutely modulate the gating of Kv4/KChIP channel complexes.

## Outlook

A more far-reaching question to be answered is whether both Kv4/KChIP/Cav nanodomain signaling and KChIP functions related to presenilins and/or to DNA may synergistically coexist in the same cell. One may ask, for instance: Can Ca^2+^ control both Kv4/KChIP/DPP channel function and the transcription of the prodynorphin gene in nociceptive neurons, or can Ca^2+^ control both Kv4/KChIP/DPP channel function and NCX3 expression in cerebellar neurons? Alternatively, KChIPs may fulfill one function under physiological and a different one under pathophysiological conditions. It is also intriguing that KChIPs themselves may be able to influence relevant sources of Ca^2+^, by controlling calcium channel gene transcription or ER Ca^2+^ release via presenilin. We need to know whether and how KChIPs are involved in different Ca^2+^ signaling mechanisms, and whether different mechanisms converge to fulfill the same physiologic function, maybe to fine-tune Kv4 channel gating. On the other hand, the role of KChIPs as neuronal and non-neuronal calcium sensors under conditions with disturbed Ca^2+^ signaling, like apoptosis or cardiac stress, has to be defined more clearly in the future.
